# Two cases of ulcerative dermatomyositis with negative antimelanoma differentiation-associated gene 5 antibody

**DOI:** 10.1016/j.jdcr.2025.02.046

**Published:** 2025-04-03

**Authors:** Janet Choi, Hatice Zengin, Bijal Amin, Benedict Wu

**Affiliations:** a Division of Dermatology, Albert Einstein College of Medicine/Montefiore Medical Center, Bronx, New York; b Department of Pathology, Albert Einstein College of Medicine/Montefiore Medical Center, Bronx, New York

**Keywords:** anti-MDA-5 negative dermatomyositis, clinically amyopathic dermatomyositis, connective tissue disease, interstitial lung disease, ulcers

*To the Editor:* We greatly appreciated Ozer et al’s unique case of antimelanoma differentiation-associated gene 5 (MDA-5) antibody-negative dermatomyositis (DM) presenting without vasculitic ulcerations or interstitial lung disease (ILD).^1^ Ulcerations are unusual in DM but may present overlying Gottron's papules (GP) or sign (GS) in the anti-MDA-5 subtype.^2^ This subtype is clinically amyopathic with associated ILD.^2^^,^^3^ Herein, we describe 2 additional cases of ulcerative MDA-5 negative DM (Table I).

**Table I:** Clinical features, serologies, autoimmune profile, and punch biopsies of 2 cases of anti-MDA-5 negative dermatomyositis

Patient	Clinical manifestations	ANA	ENA	Myositis panel∗	CK	Aldolase	Punch biopsy	Other tests
1	Violaceous and hyperpigmented painful ulcers and plaques overlying Gottron’s sign† on the elbows, inverse Gottron’s sign‡ Gottron’s papules,§ periungual telangiectasias, heliotrope rash,‖ and Samitz’s sign¶	Negative	Negative	Anti-Ro52 kD: 23 units (weak positive)	22 U/L (*N*: <200 U/L)	4.7 U/L (*N*: ≤8.1 U/L)	Subtle interface dermatitis compatible with a manifestation of collagen vascular disease, telangiectasias and postinflammatory pigmentary alteration	CT chest: ILD
2	Violaceous ulcers overlying Gottron’s sign† on the elbows and knees, Gottron’s papules,§ mucocutaneous ulcers of the lip and tongue, periungual erythema, and shawl sign#	Negative	Negative	Anti-U1 RNP: 26 units (weak positive)	112 U/L (*N*: <200 U/L)	7.1 U/L (*N*: ≤8.1 U/L)	Interface dermatitis compatible with a manifestation of collagen vascular disease, telangiectasias, and dermal alteration	CT chest/abdomen/pelvis: No evidence of ILD

*ANA*, Antinuclear antibodies; *CK*, creatine kinase; *CT*, computed tomography; *ENA*, extractable nuclear antigens; *ILD*, interstitial lung disease; *N*, normal.

∗ Myomarker 3 plus profile, consisting of: Anti-Jo-1, anti-PL-7, anti-PL12, anti-EJ, anti-OJ, anti-SRP, anti-Mi-2, anti-TIF-1gamma, anti-MDA-5, anti-NXP-2, anti-SAE1, anti-PM/Scl-100, anti-Ku, anti-Rho52, anti-U1, anti-U2, and anti-U3.

† Erythematous to violaceous macules or patches on the extensor surfaces of joints in the extremities, particularly the metacarpophalangeal, distal interphalangeal, proximal interphalangeal joints, elbows, and/or knees.

‡ Erythematous to violaceous macules or patches on the flexural surfaces of the metacarpophalangeal, distal interphalangeal, and/or proximal interphalangeal joints, characteristic of anti-MDA-5 positive dermatomyositis.

§ Erythematous to violaceous papules on the extensor surfaces of joints in the extremities, particularly the metacarpophalangeal, distal interphalangeal, proximal interphalangeal joints, elbows, and/or knees.

‖ Periorbital edema and telangiectasis.

¶ Ragged cuticles.

# Erythema of the upper back, shoulders, and posterior neck.

Patient 1 is a 37-year-old man presenting with lung failure and violaceous ulcers and plaques overlying GS on the elbows; he had GP, inverse GS, periungual telangiectasias, heliotrope rash, and Samitz’s sign (Fig 1, *A* and *B*). Antinuclear antibody was negative, but anti-Ro52 antibody was positive; aldolase and creatine kinase were not elevated. Chest computed tomography showed ILD. Punch biopsies revealed subtle interface dermatitis compatible with collagen vascular disease (Fig 2).

**Fig 1:**
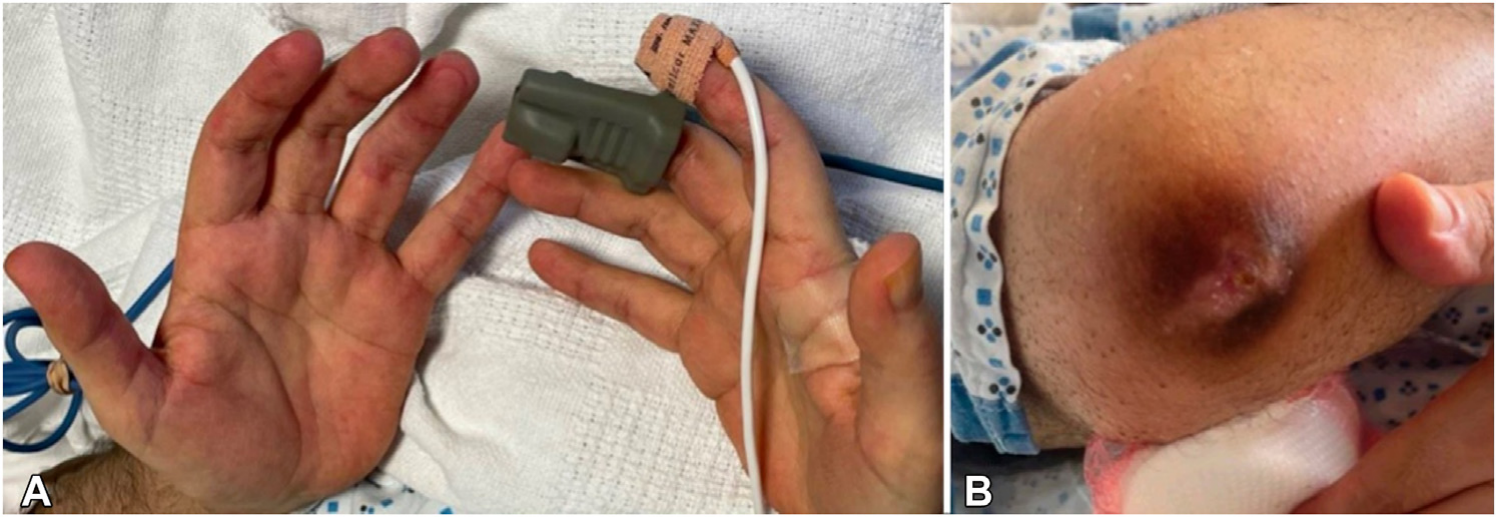
Patient 1: Clinical findings of anti-MDA-5 negative dermatomyositis. **A,** Inverse Gottron’s sign on the palms. **B,** Violaceous ulcers and plaques overlying Gottron’s sign on the elbows.

**Fig 2:**
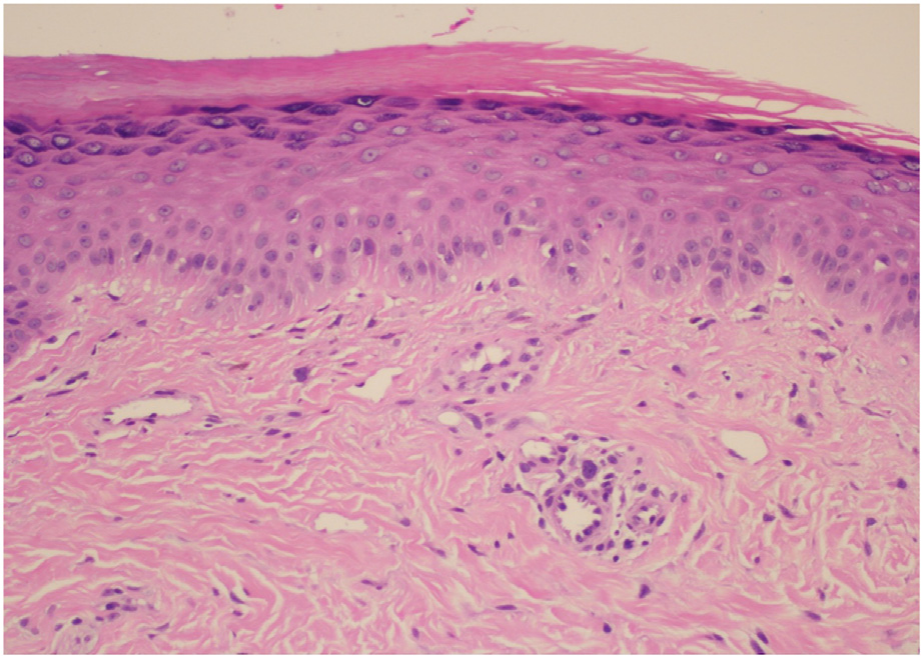
Patient 1: Histopathologic findings of anti-MDA-5 negative dermatomyositis. H&E, 20×. This image demonstrates vacuolar interface dermatitis with rare apoptotic keratinocytes. Scattered extravasated red blood cells and melanophages are present in the dermis.

Patient 2 is an 84-year-old man presenting with Raynaud’s phenomenon and worsening violaceous ulcers overlying GS on the elbows and knees, oral ulcers, GP, periungual erythema, and shawl sign (Fig 3, *A* and *B*). Serology showed negative antinuclear antibody but positive U1RNP, with nonelevated aldolase and creatine kinase. Chest computed tomography showed no ILD; punch biopsies showed interface dermatitis compatible with collagen vascular disease (Fig 4).

**Fig 3:**
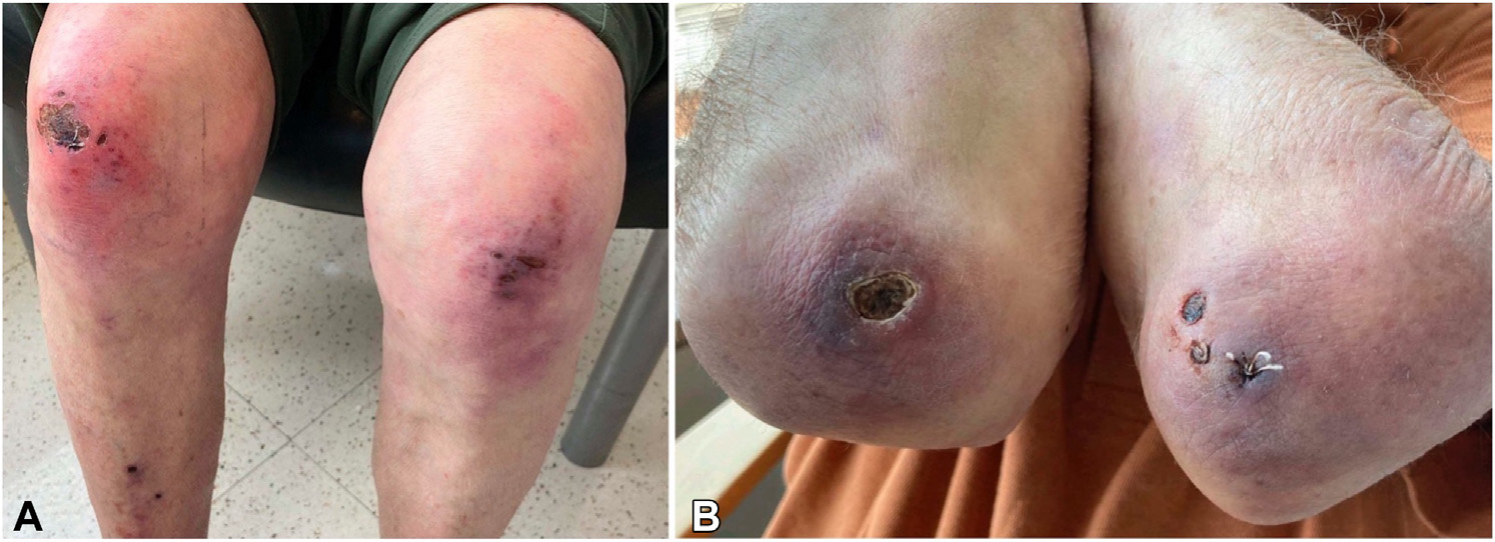
Patient 2: Clinical findings. **A,** Ulcers overlying Gottron’s sign on the knees. **B,** Ulcers overlying Gottron’s sign on the elbows.

**Fig 4:**
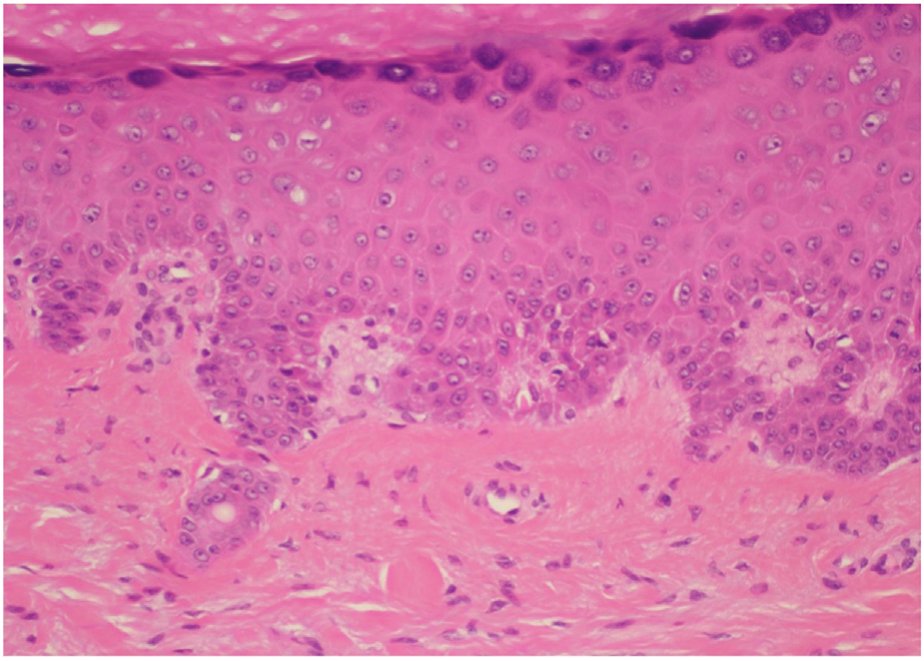
Patient 2: Histopathology. H&E, 20×. A high-power image shows interface dermatitis with scattered necrotic keratinocytes.

Although ulcers are associated with MDA-5 DM and ILD, the Ro52 and U1RNP autoantibodies are associated with ILD in various CVDs.^4^^,^^5^ Our cases emphasize the critical role of applying clinicopathologic-serologic correlation in diagnosing challenging cases of DM.

## Conflicts of interest

None disclosed.
